# Different BD BACTEC^™^ Blood Culture Bottle Types for the Detection of Fungi in Simulated Sterile Body Fluid Samples

**DOI:** 10.3390/diagnostics13101699

**Published:** 2023-05-11

**Authors:** Rok Tomazin, Tadej Pliberšek, Anja Oštrbenk Valenčak, Tadeja Matos

**Affiliations:** 1 Institute of Microbiology and Immunology, Faculty of Medicine, University of Ljubljana, Zaloška Cesta 4, SI-1000 Ljubljana, Slovenia; rok.tomazin@mf.uni-lj.si (R.T.); anja.ostrbenk@mf.uni-lj.si (A.O.V.); 2 Avacone AG, Gewerbestrasse 4, 4450 Sissach, Switzerland; tadej.plibersek@avacone.com

**Keywords:** BD BACTEC Mycosis-IC/F, BD BACTEC Plus Aerobic/F, fungal infections, blood cultures, *Candida*, *Aspergillus*, *Cryptococcus*

## Abstract

Blood culture systems are a potential alternative to classical cultivation of fungi on mycological media, but there are limited data on the suitability of these systems for culturing other sample types (e.g., sterile body fluids). We conducted a prospective study to evaluate different types of blood culture (BC) bottles for the detection of different fungal species in non-blood samples. A total of 43 fungal isolates were tested for their ability to grow in BD BACTEC Mycosis-IC/F (Mycosis bottles), BD BACTEC Plus Aerobic/F (Aerobic bottles) and BD BACTEC Plus Anaerobic/F (Anaerobic bottles) (Becton Dickinson, East Rutherford, NJ, USA) BC bottles inoculated with spiked samples without the addition of blood or fastidious organism supplement. Time to detection (TTD) was determined for all BC types tested and compared between groups. In general, Mycosis and Aerobic bottles were similar (*p* > 0.05). The Anaerobic bottles failed to support growth in >86% of cases. The Mycosis bottles were superior in detecting *Candida glabrata*, *Cryptococcus* spp. and *Aspergillus* spp. (*p* < 0.05). The performance of Mycosis and Aerobic bottles was similar, but if cryptococcosis or aspergillosis is suspected, the use of Mycosis bottles is recommended. Anaerobic bottles are not recommended for fungal detection.

## 1. Introduction

Due to advances in medicine in recent decades, which allow longer life expectancy for patients with immunodeficiency or damaged anatomical barriers, invasive fungal infections (IFIs) are becoming increasingly common [1]. Based on phylogenetic studies, we estimate that there are more than 2,000,000 fungal species, of which about 300 cause infections in humans [1,2,3]. IFIs are most commonly caused by the genera *Candida*, *Aspergillus*, *Cryptococcus* and *Pneumocystis*, which infect approximately 2,000,000 people each year and are responsible for more than 90% of all deaths attributable to fungal infections [3,4,5,6].

Despite efforts to include molecular tests in diagnostic guidelines for IFI, culturing fungi on mycological media and blood cultures remains the gold standard when fungaemia is suspected [7]. Among molecular methods, only Pneumocystis PCR and Aspergillus PCR have been included in diagnostic guidelines since 2016 and 2017, respectively [8,9]. Molecular diagnostics are also being developed for other types of IFI, which will hopefully make an important contribution to reducing morbidity and mortality. Nevertheless, culture remains the primary diagnostic approach and is the only one that allows subsequent antifungal susceptibility testing. Such testing is a very important tool for clinicians in selecting the most appropriate antifungal agent and in antifungal stewardship for optimal therapeutic decisions and patient outcomes [10]. In clinical practice, BacT/ALERT^®^ (bioMérieux, Marcy-l’Étoile, France) and BD BACTEC^TM^ (Becton Dickinson, East Rutherford, NJ, USA) blood culture (BC) systems are used to isolate bacterial and fungal pathogens from blood. Although they are primarily used to diagnose candidiasis, these systems can also be used to detect other IFIs that are diagnostically more complex than candidiasis and are caused by a broad spectrum of pathogenic fungi [11,12]. In most cases, patients suffering from IFI already have severe underlying disease, which often makes it difficult to obtain an optimal clinical sample from primarily sterile anatomical sites. The sample size is usually very small, which limits the number of examinations. BC systems have so far only been validated for the cultivation of blood samples, and there are few data on the suitability of these systems for the cultivation of other types of samples for diagnostic purposes. A few studies have shown that BC systems can be useful for the cultivation of primary sterile body fluids (SBF) other than blood, such as cerebrospinal fluid (CSF), peritoneal and synovial fluid, and pleural effusion [13,14,15]. Most studies focus on BC systems for the detection of bacterial pathogens, and therefore very little is known about their use for the diagnosis of IFIs [13,14,15,16].

The aim of this study was to test the analytical sensitivity and specificity for the detection of a broad spectrum of fungi from simulated primary sterile body fluids other than blood in BD BACTEC Mycosis-IC/F, BD BACTEC Plus Aerobic/F and BD BACTEC Plus Anaerobic/F (Becton Dickinson, USA) blood culture bottles and to compare the time to detection (TTD) between the different bottle types.

## 2. Materials and Methods

In total, 43 fungal isolates were tested for their ability to grow in three different types of blood culture bottles without blood or fastidious organism supplement added.

### 2.1. Fungal Isolates

A prospective laboratory study was conducted over a period of 5 months (March–August 2018) in the Laboratory for Diagnostics of Fungal Infections, Institute of Microbiology and Immunology, Faculty of Medicine, University of Ljubljana, Ljubljana, Slovenia. A total of 43 fungal isolates were obtained from various clinical samples that were processed in routine diagnostics. The following 25 different fungal species with a corresponding number of isolates were included in the study: *Aspergillus fumigatus* (*n* = 2), *Aspergillus lentulus* (*n* = 1), *Aspergillus niger* (*n* = 2), *Aspergillus terreus* (*n* = 2), *Aspergillus ustus* (*n* = 3), *Candida albicans* (*n* = 2), *Candida dubliniensis* (*n* = 1), *Candida glabrata* (*n* = 2), *Candida parapsilosis* (*n* = 2), *Candida tropicalis* (*n* = 2), *Clavispora lusitaniae* (*n* = 2), *Cryptococcus deneoformans* (*n* = 2), *Cryptococcus neoformans* (*n* = 2), *Cunninghamella bertholletiae* (*n* = 1), *Exophiala dermatitidis* (*n* = 2), *Fusarium* sp. (*n* = 1), *Lichtheimia corymbifera* (*n* = 1), *Magnusiomyces capitatus* (*n* = 2), *Meyerozyma guilliermondii* (*n* = 1), *Mucor* sp. (*n* = 1), *Paecilomyces variotii* (*n* = 1), *Purpureocillium lilacinum* (*n* = 1), *Rasamsonia argillacea* (*n* = 3), *Saprochaete clavata* (*n* = 2) and *Scedosporium apiospermum* (*n* = 2). The species included in the study were selected from isolates isolated from clinical material received in the five-month collection period. Most of these data come from analyses of data from laboratory information systems and are not yet ready for full publication. *Candida albicans* ATCC 90028, *Candida parapsilosis* ATCC 22019 and *Candida krusei* ATCC 6285 were used as controls.

### 2.2. Simulated Primary Sterile Body Fluids

Before spiking the samples, the fungal cultures were grown for 24 h on Sabouraud agar supplemented with gentamycin and chloramphenicol (bioMérieux, Marcy-l’Étoile, France). Fungal cultures were suspended in sterile saline (0.9% NaCl), used as an analogue to the primary sterile body fluid, to achieve a standard density of 0.5 McFarland (equivalent to approximately 10^6^ CFU/mL). A serial dilution was prepared in 8 mL to achieve concentrations of inoculum of 10 CFU/mL and 100 CFU/mL, which were then inoculated into (i) BD BACTEC-Mycosis IC/F, (ii) BD BACTEC Aerobic Plus/F and (iii) BD BACTEC Anaerobic Plus/F (Becton Dickinson, NJ, USA). Each fungal isolate was inoculated into all three types of blood culture bottles in two different inocula in duplicate: 10 CFU/mL and 100 CFU/mL. For quantification and re-identification, 0.2 mL of each serial dilution was also inoculated onto Sabouraud agar and incubated overnight at 37 °C. The next day, all strains growing on Sabouraud agar were quantified (CFU/mL), and identified by their morphological characteristics [17] and using MALDI-TOF MS (Bruker Daltonik, Bremen, Germany). In brief, a single colony (yeasts) or a piece of mycelium (moulds) was spread on the MALDI steel plate and overlaid with 1 µL of 98% formic acid. After drying, the sample was overcoated with 1 µL of photoabsorbent-saturated α-cyano-4-hydroxycinnamic acid (HCCA) matrix solution in 50% acetonitrile–2.5% trifluoroacetic acid (Bruker Daltonik, Germany) and dried before subsequent analysis using a Linear-Mode microflex LT/SH MALDI-TOF MS system, Biotyper RTC software version 3.1 (Bruker Daltonik). Identifications with a score value of ≥1.70 were considered reliable. The Bruker bacterial test standard (Bruker Daltonik) was used for calibration according to the manufacturer’s instructions.

### 2.3. Incubation, Time to Detection and Contamination Control

After inoculation, the blood culture bottles were placed in the BD BACTEC^TM^ FX system (BD, USA) for incubation and automatic detection of positivity. The total incubation period was 5 days for BD BACTEC Aerobic Plus/F and BD BACTEC Anaerobic Plus/F, and 14 days for BD BACTEC-Mycosis IC/F, as recommended by the manufacturer. If the bottle was not positive during this incubation period, it was considered negative. Time to detection (TTD) was recorded and bottles were checked for contamination by inoculation on Sabouraud agar: After 24 h incubation at 35 °C, fungi that grew were identified by their morphological characteristics [17] and with MALDI-TOF MS (Bruker Daltonik, Germany). We examined the distribution of TTD, measured in hours, for fungal isolates classified into three different groups: *Candida* spp., *Cryptococcus* spp. and other yeast species, and *Aspergillus* spp. and other filamentous fungi.

### 2.4. Statistical Analysis

An unpaired two-sample *t*-test was used to determine the correlation between the average TTDs of the different BC bottles. All data analyses were performed using R software version 3.5.1 (Free Software Foundation, Boston, MA, USA), and a *p*-value of <0.05 was considered statistically significant.

## 3. Results

**Figure 1:**
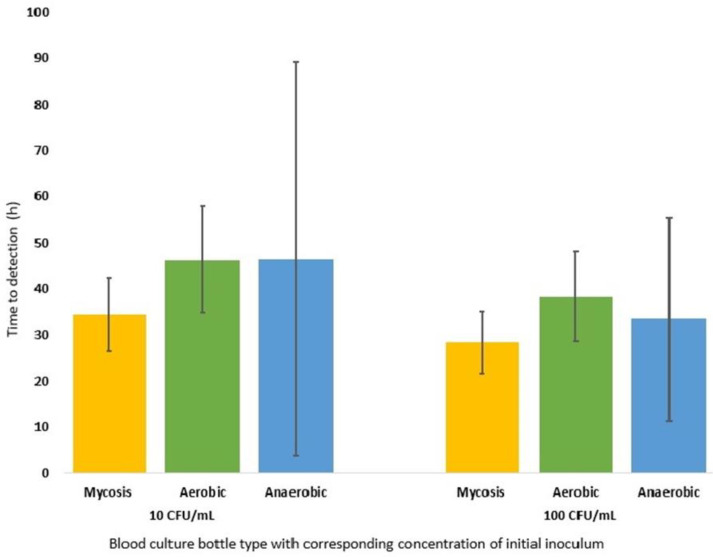
Average time to detection (TTD) with 95% confidence intervals for 43 fungal isolates based on different inocula and blood culture systems: BD BACTEC-Mycosis IC/F (Mycosis, in yellow), BD BACTEC Aerobic Plus/F (Aerobic, in green) and BD BACTEC Anaerobic Plus/F (Anaerobic, in blue).

Positivity rates for BACTEC-Mycosis IC/F bottles inoculated with low (10 CFU/mL) and high (100 CFU/mL) inoculum were 42/43 (97.7%) and 43/43 (100.0%), respectively. The number of positive BACTEC Plus Aerobic/F vials was similar, with positivity rates of 40/43 (93.0%) for low inoculum and 41/43 (95.3%) for high inoculum. In contrast, with BACTEC Plus Anaerobic/F BD BACTEC growth was only detected in 5/43 (11.6%) bottles with low inoculum and 6/43 (14.0%) bottles with high inoculum. All five and six positive BACTEC Plus Anaerobic/F BD BACTEC vials belonged to the *Candida* spp. group. Due to their low sensitivity, the BACTEC Plus Anaerobic/F BD BACTEC blood culture bottles were excluded from further analysis. The average TTDs of all fungal isolates and inoculum categories are shown in Figure 1. The BD BACTEC-Mycosis IC/F had a slightly better TTD compared to BD BACTEC Aerobic Plus/F and BD BACTEC Anaerobic Plus/F; nevertheless, no statistically significant difference was found in the detection of growth (all *p*-values > 0.05).

### 3.1. Detecting Candida *spp.*

BACTEC Mycosis-IC/F vials detected all (12/12) fungal isolates of *Candida* spp. regardless of the initial inoculum amount, while BACTEC Plus Aerobic/F missed one strain of *C. glabrata* at both inoculum amounts. The average TTDs with 95% confidence interval for *Candida* spp. based on different inocula and blood culture systems are shown in Table 1 and were lower on Mycosis than on Aerobic media, especially for *C. glabrata* (15.74 h vs. 71.75 h for low inoculum and 12.19 h vs. 44.97 h for medium inoculum).

### 3.2. Detecting Cryptococcus *spp.* and Other Yeast Species

The detection rate of *Cryptococcus* spp. and other yeast species was 100% in BACTEC Mycosis-IC/F in both inocula and 90.0% in BACTEC Plus Aerobic/F, while one strain of *C. deneoformans* was not detected in either inoculum by Aerobic media. For fungal isolates belonging to *Cryptococcus* spp. and *Exophiala dermatitidis*, the average TTD on Mycosis media was lower compared to Aerobic media and reached statistical significance (Table 2 and Table 3).

### 3.3. Detecting Aspergillus *spp.* and Other Moulds

Of 21 fungal isolates belonging to *Aspergillus* spp. and other moulds, we were able to assess growth in 19 blood culture vials. All (19/19) BACTEC Mycosis-IC/F and BACTEC Plus Aerobic/F vials showed a positive result. As shown in Table 4, we did not find statistically significant differences in the average TTDs between the different blood culture vials. However, when we analysed only the fungal isolates belonging to *Aspergillus* spp., growth was found statistically significantly earlier in medium inoculum (19.99 h vs. 24.89 h; *p* = 0.027) in Mycosis compared to Aerobic (Table 5). Interestingly, TTDs for fungal isolates belonging to *Rasamsonia argillacea* were detected much earlier at low inoculum (65.80 h vs. 111.54 h) and high inoculum (56.47 h vs. 99.93 h) in Aerobic than in Mycosis; however, only three strains were tested.

## 4. Discussion

BC systems are used in clinical microbiology laboratories for detecting bacteraemia, fungaemia, and pathogenic bacteria and fungi in SBFs. BD BACTEC Plus Aerobic/F, BD BACTEC Plus Anaerobic/F and BD BACTEC Mycosis-I/F are intended for the rapid detection of bacteria and fungi in blood (Becton Dickinson, NJ, USA). BACTEC Plus Aerobic/F is an all-purpose, enriched soybean–casein digest broth medium that supports the growth of aerobic organisms. This medium contains resins for antibiotic neutralization, while BD BACTEC Plus Anaerobic/F is an anaerobic medium that facilitates the detection and recovery of anaerobes. It contains a detergent to lyse both red and white blood cells in the sample, releasing all intracellular organisms. The third BC bottle type used was BD BACTEC Mycosis-I/F—a complex medium used for selective culture and recovery of fungi from blood samples. The bottle contains brain–heart infusion enriched with sucrose (Becton Dickinson, NJ, USA). It also contains tobramycin and chloramphenicol to suppress bacterial growth and saponin to release phagocytosed fungi from leukocytes (Becton Dickinson, NJ, USA). BD BACTEC Mycosis-I/F alone costs about 2.2 times as much as the bottles of standard aerobic or anaerobic medium. Because of this limitation, many hospitals do not routinely use dedicated mycology media for blood cultures. Besides blood, BC systems can also be used for other sample types, as shown in several studies [14,18,19,20,21]. A study by Çetin et al. showed a statistically significantly higher sensitivity for detecting pathogenic agents in SBFs using the BD BACTEC BC system in comparison with conventional microbiological culture methods (*p* < 0.0001) [14,18]. The greatest advantage of BC systems is higher sensitivity for detecting slow-growing or fastidious bacteria such as *Brucella melitensis*, *Streptococcus pneumoniae*, *Neisseria meningitidis*, *Pseudomonas fluorescens* and *Rothia dentocariosa*, particularly from SBFs such as cerebrospinal fluid and synovial fluid, compared to classical culture. The study also showed a statistically significant shorter TTD for the BC system (*p* < 0.001) [14].

Recently, several studies have evaluated the use of BC systems for detecting pathogenic agents from sonicated retrieved orthopaedic implants and ophthalmic samples [19,21]. However, less is known about detecting pathogenic fungi from SBFs with BC systems. Clinical samples that could benefit from this approach are SBFs such as CSF, vitreous, peritoneal and synovial fluid, pleural effusion, drainage fluid and abscess aspirates—samples in which both bacterial and fungal pathogens can be expected, but the quantity of which is usually too small for classical inoculation of many bacteriological and mycological media. Bronchoalveolar lavage samples could also benefit from this approach to some extent, especially if transport to the microbiology laboratory is likely to take too long. The problem here, however, could be overgrowth by the upper respiratory tract microbiota if the samples are not collected quite correctly. Many species of fungi can grow from such a variety of clinical specimens—it is therefore important to know which fungi can actually be detected by BC systems. In Slovenia, similarly to other European countries, *Candida* spp. and *Aspergillus* spp. are the most common causes of invasive fungal infections, accounting for more than 90% of all isolates [22,23,24]. Other fungi such as *Mucorales*, *Cryptococcus* spp., *Fusarium* spp., *Scedosporium* spp. and *Paecilomyces* spp. that could be detected using this approach are less common [25,26,27]. A review of the literature reveals that there are limited data on using BC systems for detecting pathogenic fungi in SBFs, with most studies focusing only on *Candida* spp. [28,29,30,31]. Our study evaluated the BD BACTEC system for isolating a wide variety of medically important fungi from simulated SBFs. We included not only *Candida* spp., but also members of *Cryptococcus* spp., *Aspergillus* spp., *Mucorales*, and some other fungal species that are less common in the clinical setting. In total, we conducted 43 experiments in duplicate with concentrations of 10 CFU/mL and 100 CFU/mL of initial inoculum in simulated SBFs. Some medically important fungi such as *Candida auris*, *Candida krusei* and *Trichosporon asahii* were unfortunately not included because we did not isolate them from the clinical material during the five-month observation period. We tried to include as many different fungal species as possible, which we succeeded in doing, but there were only one to three isolates per species, so the results are unfortunately not statistically relevant for each species. The sensitivity of BD BACTE Mycosis IC/F and Plus Aerobic/F bottles was 100% and 95.3%, respectively. This is an important observation for two main reasons: first, both BC bottle types—bacteriological and mycological—can detect a wide variety of medically important fungi without the addition of supplements, and, second, both have high sensitivity. In the clinical setting there are many cases when the quantity of the patient sample is not sufficient for several microbiological investigations, thus using just one bottle type for detecting both bacteria and fungi can reduce the need to divide the sample between different laboratories. Using a BC system is superior to standard culture on agarized media because it is a closed system with a lower risk of contamination. The drawback of this study is that actual clinical samples were not included so a direct comparison between simulated and real clinical samples was not possible. The study did show, however, that detection of a wide variety of fungal species using BC bottles is possible even without the addition of supplements, in contrast to Nylen et al. [32], who primarily studied the addition of supplements in growth media of BC bottles to evaluate the isolation rate. The study by Nylen et al. showed that the BACTEC-Mycosis IC/F bottle is more appropriate for *Candida* isolation than BD BACTEC Aerobic/Anaerobic bottles, which is in agreement with our data.

Our study shows a shorter TTD of BD BACTEC-Mycosis IC/F bottles in comparison with BD BACTEC Plus Aerobic/F. The differences in the TTD between bottles were up to 10 min for detecting filamentous fungi, and up to 36 h for detecting *Cryptococcus* spp. and other non-*Candida* yeasts. In the case of non-*Candida* yeast species, the difference in the TTD is statistically significant (*p* = 0.046), especially for cryptococci (*p* = 0.002), and therefore we strongly recommend BD BACTEC Mycosis IC/F when disseminated cryptococcosis is suspected. These findings are in alignment with some other studies that also show a shorter TTD with the BD BACTEC-Mycosis IC/F bottle for *Cryptococcus neoformans* as well as for *C. glabrata*, *C. albicans* and *C. parapsilosis* [28,29,30]. Our research also shows a longer TTD for BD BACTEC Plus Aerobic/F in the case of *C. glabrata*, but not for *Candida* spp. in general. Other studies have found that mycological bottle types have higher sensitivity than bacteriological ones [30,31], even when antifungal treatment is given, but the overall level of detection of episodes leading to revised antifungal therapy does not differ in practice. Our data and others argue for using both types of BC bottles to maximise the detection rate of candidaemia and bacteraemia [30,31]. Our data did not show statistically significant differences in the TTD for the filamentous-fungi group (*p* = 0.99), but when examining data for *Aspergillus* spp. alone at the initial inoculum concentration of 100 CFU/mL we noticed a shorter TTD (4.6 h) when using the BD BACTEC-Mycosis IC/F bottle rather than BD BACTEC Plus Aerobic/F bottles (*p* = 0.027). On the other hand, Rosa et al. [33] did not find differences in the TTD between BD BACTEC-Mycosis IC/F and Plus Aerobic/F bottles for detecting aspergilli. BD BACTEC Anaerobic Plus/F bottles did not turn out to be suitable for detecting any of the filamentous fungi tested. None of the experiments could provide a positive signal of growth in the BC bottles mentioned. In contrast to aspergilli, the TTD of *Mucorales*, *Rasamsonia argillacea* and *Purpureocillium lilacinium* was shorter with the BD BACTEC Plus Aerobic/F bottle than with BD BACTEC-Mycosis IC/F. Unfortunately, the number of tests and strains included was low, which offers the opportunity for similar extended studies that can provide more data. Additionally, the TTD using a classical mycological approach (culture of samples on agarized media) could not be precisely calculated because the inoculated Sabouraud plates were checked only after an overnight incubation and were not monitored at 10 min intervals as the BC bottles were. The bottles were not subjected to an extended incubation time, which is one of the limitations of this study—the incubation time was limited by the manufacturer’s recommendations. If the negative bottles had been re-incubated with rescanning, the number of positive bottles might have increased.

BD BACTEC Plus Anaerobic/F BCs for detecting fungi in SBFs have proven inefficient: sensitivity was 13.9% when the inoculum concentration was 100 CFU/mL and 11.6% when the inoculum concentration was 10 CFU/mL. The low detection rate can be explained by the fact that *Candida* spp. are facultative anaerobes and have longer generation times in anaerobic conditions, thus not growing sufficiently to be detected by BD BACTEC Plus Anaerobic/F [34]. Growth in BC bottles depends on the metabolic capacity of the microbe, so the chemical composition of the medium and atmosphere is crucial for successful detection. Therefore, there are different types of bottles that better support a particular metabolic type of microorganism, e.g., anaerobic bottles allow the growth of anaerobes and facultative anaerobes, while aerobic bottles favour the growth of strict aerobes and facultative anaerobes, and Mycosis bottles allow the growth of fungi with the addition of antibiotics to suppress bacterial growth. Medically important fungi obtain their energy either by fermentation (e.g., *Saccharomyces*), aerobic oxidation (e.g., *Cryptococcus* and *Yarrowia*) or the combination of both, which predominates [35,36]. The Crabtree effect is also interesting for their metabolism: despite the presence of the respiratory chain, some species gain ATP even under aerobic conditions mainly by fermentation rather than oxidative phosphorylation [35,36]. Fungi are therefore relatively undemanding organisms to culture, as they can obtain energy from a wide range of biomolecules—from simple sugars to complex polysaccharides, proteins and lipids. However, they thrive best under aerobic conditions, as our study shows, because aerobic BC bottles were able to grow the most isolates, while anaerobic BC bottles supported growth in less than 14%. Our research showed that the sensitivity of both BD BACTEC-Mycosis IC/F and BD BACTEC Aerobic/F bottles is high and that both are appropriate for detecting fungi in SBFs. However, it would be good to perform an extended study with more isolates of each species to more reliably determine the sensitivity of the method for each species or group of microorganisms. BD BACTEC Aerobic/F bottles are also appropriate for cultivating samples when bacterial pathogens are suspected in addition to fungi. Using BC systems decreases contamination rates of samples to a minimum. Our study has shown that, when disseminated aspergillosis or cryptococcosis is suspected, BD BACTEC-Mycosis IC/F is more appropriate for detecting *Aspergillus* spp. and *Cryptococcus* spp. in the patient’s blood due to a short TTD. This can be crucial for managing critically ill patients. Because of poor sensitivity, we do not recommend using BD BACTEC Anaerobic/F bottles for detecting pathogenic fungi in SBFs.

## 5. Conclusions

In this study, we found that BD BACTEC-Mycosis IC/F and BD BACTEC Aerobic/F bottles can detect various fungal species in 95.3% to 100% of cases, even without the addition of growth supplements. BD BACTEC-Mycosis IC/F has been shown to be particularly helpful in recovering *Aspergillus* spp. and *Cryptococcus* spp. due to its high sensitivity and shorter time to detection compared to BD BACTEC Aerobic/F. BD BACTEC Anaerobic/F bottles have not proven to be an optimal choice when invasive mycoses are suspected, as they do not support fungal growth in at least 86.1% of cases. Based on our findings, we recommend BD BACTEC-Mycosis IC/F and BD BACTEC Aerobic/F bottles for primarily sterile clinical fluid samples in suspected invasive mycoses.

## Figures and Tables

**Table 1:** Average time to detection (TTD, in hours) of *Candida* spp. with BACTEC Mycosis-IC/F and BACTEC Plus Aerobic/F.

Initial Inoculum (CFU/mL)	Blood Culture Bottle Type	Average Time to Detection TTD (h)	95% Confidence Interval	*p*-Value
10	Mycosis	17.55	14.88–20.22	0.095
	Aerobic	25.82	15.16–36.47	
100	Mycosis	14.57	12.67–16.47	0.082
	Aerobic	19.63	13.51–25.74	

**Table 2:** Average time to detection (TTD, in hours) of *Cryptococcus* spp. and other yeast species with BACTEC Mycosis-IC/F and BACTEC Plus Aerobic/F.

Initial Inoculum (CFU/mL)	Blood Culture Bottle Type	Average Time to Detection TTD (h)	95% Confidence Interval	*p*-Value
10	Mycosis	34.35	24.08–44.62	0.046
	Aerobic	74.01	30.42–117.59	
100	Mycosis	27.78	19.72–35.84	0.044
	Aerobic	62.55	24.51–100.59	

**Table 3:** Average time to detection (TTD, in hours) of *Cryptococcus* spp. with BACTEC Mycosis-IC/F and BACTEC Plus Aerobic/F.

Initial Inoculum (CFU/mL)	Blood Culture Bottle Type	Average Time to Detection TTD (h)	95% Confidence Interval	*p*-Value
10	Mycosis	41.08	24.58–57.58	0.002
	Aerobic	137.58	64.72–210.98	
100	Mycosis	34.1	19.53–48.66	0.001
	Aerobic	119.4	61.07–177.74	

**Table 4:** Average time to detection (TTD, in hours) of *Aspergillus* spp. and other moulds with BACTEC Mycosis-IC/F and BACTEC Plus Aerobic/F.

Initial Inoculum (CFU/mL)	Blood Culture Bottle Type	Average Time to Detection TTD (h)	95% Confidence Interval	*p*-Value
10	Mycosis	44.34	29.22–59.46	0.99
	Aerobic	44.44	32.43–56.45	
100	Mycosis	36.11	22.88–49.34	0.90
	Aerobic	37.09	27.07–47.12	

**Table 5:** Average time to detection (TTD, in hours) of *Aspergillus* spp. with BACTEC Mycosis-IC/F and BACTEC Plus Aerobic/F.

Initial Inoculum (CFU/mL)	Blood Culture Bottle Type	Average Time to Detection TTD (h)	95% Confidence Interval	*p*-Value
10	Mycosis	24.23	21.79–26.67	0.092
	Aerobic	28.86	23.18–34.55	
100	Mycosis	19.99	18.71–21.26	0.027
	Aerobic	24.89	20.45–29.33	

## Data Availability

For research data please contact Tadeja Matos, tadeja.matos@mf.uni-lj.si.

## References

[1] Vallabhaneni S., Mody R.K., Walker T., Chiller T. (2016). The global burden of fungal diseases. Infect. Dis. Clin. N. Am..

[2] Schmiedel Y., Zimmerli S. (2016). Common invasive fungal diseases: An overview of invasive candidiasis, aspergillosis, cryptococcosis, and Pneumocystis pneumonia. Swiss Med Wkly..

[3] Hawksworth D.L., Lücking R. (2017). Fungal diversity revisited: 2.2 to 3.8 million species. Microbiol. Spectr..

[4] Medina N., Samayoa B., Lau-Bonilla D., Denning D., Herrera R., Mercado D., Guzmán B., Pérez J.C., Arathoon E. (2017). Burden of serious fungal infections in Guatemala. Eur. J. Clin. Microbiol. Infect. Dis..

[5] Gangneux J.P., Bougnoux M.E., Hennequin C., Godet C., Chandenier J., Denning D., Dupont B., LIFE program, the Société Française de Mycologie Médicale SFMM-Study Group (2016). An estimation of burden of serious fungal infections in France. J. Mycol. Med..

[6] Pegorie M., Denning D.W., Welfare W. (2016). Estimating the burden of invasive and serious fungal disease in the United Kingdom. J. Infect..

[7] Arendrup M., Boekhout T., Akova M., Meis J., Cornely O., Lortholary O. (2014). ESCMID and ECMM joint clinical guidelines for the diagnosis and management of rare invasive yeast infections. Clin. Microbiol. Infect..

[8] Alanio A., Hauser P.M., Lagrou K., Melchers W.J., Helweg-Larsen J., Matos O. (2016). ECIL guidelines for the diagnosis of *Pneumocystis jirovecii pneumonia* in patients with haematological malignancies and stem cell transplant recipients. J. Antimicrob. Chemother..

[9] Ullmann A.J., Aguado J.M., Arikan-Akdagli S., Denning D.W., Groll A.H., Lagrou K., Lass-Flörl C., Lewis R.E., Munoz P., Verweij P.E. (2018). Diagnosis and management of Aspergillus diseases: Executive summary of the 2017 ESCMID-ECMM-ERS guideline. Clin. Microbiol. Infect..

[10] Mourad A., Perfect J.R. (2017). What can the clinical mycology laboratory do for clinicians today and tomorrow?. Curr. Clin. Microbiol. Rep..

[11] Arendrup M.C., Sulim S., Holm A., Nielsen L., Nielsen S.D., Knudsen J.D., Drenck N.E., Christensen J.J., Johansen H.K. (2011). Diagnostic issues, clinical characteristics, and outcomes for patients with fungemia. J. Clin. Microbiol..

[12] Arendrup M.C., Bruun B., Christensen J.J., Fuursted K., Johansen H.K., Kjældgaard P., Knudsen J.D., Kristensen L., Møller J., Nielsen L. (2011). National surveillance of fungemia in Denmark (2004 to 2009). J. Clin. Microbiol..

[13] Bourbeau P., Riley J., Heiter B.J., Master R., Young C., Pierson C. (1998). Use of the BacT/Alert blood culture system for culture of sterile body fluids other than blood. J. Clin. Microbiol..

[14] Çetin E.S., Kaya S., Demirci M., Aridogan B.C. (2007). Comparison of the BACTEC blood culture system versus conventional methods for culture of normally sterile body fluids. Adv. Ther..

[15] Yagupsky P., Press J. (1997). Use of the isolator 1.5 microbial tube for culture of synovial fluid from patients with septic arthritis. J. Clin. Microbiol..

[16] Riedel S., Eisinger S.W., Dam L., Stamper P.D., Carroll K.C. (2011). Comparison of BD Bactec Plus Aerobic/F medium to VersaTREK Redox 1 blood culture medium for detection of Candida spp. in seeded blood culture specimens containing therapeutic levels of antifungal agents. J. Clin. Microbiol..

[17] de Hoog G.S., Guarro J., Gené J., Ahmed S.A., Al-Hatmi A.M.S., Figueras M.J., Vitale R.G. (2015). Atlas of Clinical Fungi: The Ultimate Benchtool for Diagnostics.

[18] Akcam F.Z., Yayli G., Uskun E., Kaya O., Demir C. (2006). Evaluation of the Bactec microbial detection system for culturing miscellaneous sterile body fluids. Res. Microbiol..

[19] Brown T.S., Petis S.M., Osmon D.R., Mabry T.M., Berry D.J., Hanssen A.D., Abdel M.P. (2018). Periprosthetic joint infection with fungal pathogens. J. Arthroplast..

[20] Podleska L., Lendemans S., Schmid E., Hussmann B., Nast-Kolb D., Taeger G. (2011). Sample taking during orthopedic surgery: Sensitivity and specificity using the BACTEC blood culture system. Eur. J. Clin. Microbiol. Infect. Dis..

[21] Thuret G., Carricajo A., Vautrin A.C., Raberin H., Acquart S., Garraud O., Gain P., Aubert G. (2005). Efficiency of blood culture bottles for the fungal sterility testing of corneal organ culture media. Br. J. Ophthalmol..

[22] Arendrup M.C., Fuursted K., Gahrn-Hansen B., Schønheyder H.C., Knudsen J.D., Jensen I.M., Bruun B., Christensen J.J., Johansen H.K. (2008). Semi-national surveillance of fungaemia in Denmark 2004–2006: Increasing incidence of fungaemia and numbers of isolates with reduced azole susceptibility. Clin. Microbiol. Infect..

[23] Matos T., Lejko Zupanc T., Skofljanec A., Jazbec A., Matos E., Maver Vodičar P., Germ J., Ciglar T., Tomazin R., Kofol R. (2021). Candidaemia in Central Slovenia: A 12-year retrospective survey. Mycoses.

[24] Pappas P.G., Kauffman C.A., Andes D., Benjamin D.K., Calandra T.F., Edwards J.E., Filler S.G., Fisher J.F., Kullberg B.J., Ostrosky-Zeichner L. (2009). Clinical practice guidelines for the management of candidiasis: 2009 update by the Infectious Diseases Society of America. Clin. Infect. Dis..

[25] Bassetti M., Merelli M., Righi E., Diaz-Martin A., Rosello E.M., Luzzati R., Parra A., Trecarichi E.M., Sanguinetti M., Posteraro B. (2013). Epidemiology, species distribution, antifungal susceptibility, and outcome of candidemia across five sites in Italy and Spain. J. Clin. Microbiol..

[26] Pemán J., Cantón E., Quindós G., Eraso E., Alcoba J., Guinea J., Merino P., Ruiz-Pérez-De-Pipaon M.T., Pérez-Del-Molino L., Linares-Sicilia M.J. (2012). Epidemiology, species distribution and in vitro antifungal susceptibility of fungaemia in a Spanish multicentre prospective survey. J. Antimicrob. Chemother..

[27] Prakash H., Chakrabarti A. (2019). Global Epidemiology of Mucormycosis. J. Fungi.

[28] Fricker-Hidalgo H., Chazot F., Lebeau B., Pelloux H., Ambroise-Thomas P., Grillot R. (1998). Use of simulated blood cultures to compare a specific fungal medium with a standard microorganism medium for yeast detection. Eur. J. Clin. Microbiol. Infect. Dis..

[29] Nawrot U., Kowalska-Krochmal B., Sulik-Tyszka B., Kozak M., Świętek K., Pajączkowska M., Piątkowska E., Rosiak D., Swoboda-Kopeć E. (2014). Evaluation of blood culture media for the detection of fungi. Eur. J. Clin. Microbiol. Infect. Dis..

[30] Magallon A., Basmaciyan L., Chapuis A., Valot S., Sautour M., Bador J., Dalle F. (2022). Evaluation of the relevance of use of the BD-BACTEC^®^MycosisIC/F, BD-BACTEC^®^PlusAerobic/F, BD-BACTEC^®^Lytic/10 anaerobic/F and BD-BACTEC^®^PedsPlus/F culture bottle system for fungemia detection: A 4-year retrospective study at the Dijon university hospital, France. J. Med Mycol..

[31] McDonald L.C., Weinstein M.P., Fune J., Mirrett S., Reimer L.G., Reller L.B. (2001). Controlled comparison of BacT/ALERT FAN aerobic medium and BATEC fungal blood culture medium for detection of fungemia. J. Clin. Microbiol..

[32] Nylén T., Saeedi B., Borg C., Ullberg M., Özenci V. (2013). The performance of 4 different supplements and 5 blood culture bottles types in detection of bacteria and Candida spp. in simulated sterile body fluid cultures. Diagn. Microbiol. Infect. Dis..

[33] Rosa C., Araujo R., Rodrigues A.G., Pinto-de-Sousa M.I., Pina-Vaz C. (2011). Detection of Aspergillus species in BACTEC blood cultures. J. Med Microbiol..

[34] Dumitru R., Hornby J.M., Nickerson K.W. (2004). Defined anaerobic growth medium for studying Candida albicans basic biology and resistance to eight antifungal drugs. Antimicrob. Agents Chemother..

[35] Pfeiffer T., Morley A. (2014). An evolutionary perspective on the Crabtree effect. Front. Mol. Biosci..

[36] Péter G., Rosa C.A. (2006). Biodiversity and Ecophysiology of Yeasts.

